# Efficacy and Safety of Subcutaneous Allergen-Specific Immuno-Therapy in Horses with Allergic Cutaneous and Respiratory Diseases—A Systematic Review

**DOI:** 10.3390/vetsci10100613

**Published:** 2023-10-10

**Authors:** Ina Herrmann, Adrianna Jordan Sanchez

**Affiliations:** 1 Department of Clinical Sciences, College of Veterinary Medicine, North Carolina State University, Raleigh, NC 27607, USA; 2 Department of Animal Sciences, College of Agriculture and Life Sciences, North Carolina State University, Raleigh, NC 27606, USA; ajsanche@ncsu.edu

**Keywords:** adverse events, allergy, allergen-specific immunotherapy, equine, horse

## Abstract

**Simple Summary:**

This study aimed to fill the gap in systematic reviews on the efficacy and safety of subcutaneous allergen-specific immunotherapy (AIT) in horses with allergic diseases. The review included horses with respiratory diseases, urticaria, and pruritic dermatitis treated with insect monotherapy or multi-allergen AIT. Beneficial effects were observed with multi-allergen AIT in respiratory diseases, urticaria, and pruritic dermatitis. However, when insect AIT was used solely for pruritic dermatitis, the response was less favorable. Overall, AIT demonstrated safety, with any adverse events generally being self-limiting. The review highlighted the presence of common biases and confounding factors in the included studies, warranting the need for more rigorous research.

**Abstract:**

Allergen-specific immunotherapy (AIT) is the only current intervention that has the ability to modify the immune response toward a tolerogenic state. This study aimed to assess the efficacy and safety of AIT in horses with allergic diseases in a systematic manner. Three databases were searched to identify articles reporting clinical outcomes and adverse events associated with AIT. The articles were evaluated for beneficial responses to AIT, defined as a ≥50% reduction in clinical signs, and clinical remission. Horses with respiratory diseases, urticaria, and pruritic dermatitis receiving insect monotherapy or multi-allergen AIT were included. All adverse events were graded, and analytical and confounding biases were assessed. The results showed that multi-allergen AIT had a beneficial response in 75% of horses with respiratory diseases, 88% with urticaria, and 56% with pruritic dermatitis. However, horses treated solely with insect AIT for pruritic dermatitis had a lower response rate (36%). Self-limiting local reactions were the most common adverse events, with systemic reactions grade II accounting for 11% of reported events. Analytical and confounding biases were identified as major limitations in the available studies. Further research is needed to address these biases and provide stronger evidence on the efficacy and safety of AIT in horses with allergic diseases.

## 1. Introduction

Allergic syndromes in horses can manifest with cutaneous signs such as urticaria, pruritus, and papules, or respiratory symptoms, allergic asthma, previously referred to as recurrent airway obstruction or chronic obstructive pulmonary disease [1,2]. In equine allergology, common allergens are of insect origin, often Culicoides saliva, or of environmental origin [1]. Clinical signs of allergic syndromes in horses can be alleviated with glucocorticoids and/or antihistamines [1,3]. However, horses are sensitive to the side effects of systemic glucocorticoids, and antihistamines may not effectively control the symptoms [4,5]. As a result, allergen-specific immunotherapy (AIT) is frequently employed to manage equine allergy symptoms [1,6]. AIT involves the gradual oral or subcutaneous administration of increasing amounts of allergens to an allergic subject, aiming to ameliorate the symptoms associated with subsequent exposure to the causative allergen [7]. The clinical benefit of AIT can manifest as an improvement/remission of clinical signs and/or a reduction in the need for anti-allergic medications. Despite its common clinical use and the reviewing literature describing AIT as beneficial and safe in managing equine allergic diseases [1,6], systematic reviews reporting detailed data and assessing biases are not currently available. Therefore, the objectives of this study are twofold: first, to review the evidence for the efficacy of AIT in various equine allergic syndromes, including the assessment of study biases following the PRISMA 2020 guidelines [8]; and secondly, to report the available safety information regarding AIT used to treat equine allergic diseases.

## 2. Methods

Due to the two-fold objective of the study, the results are presented in two separate sections. Part I summarizes the efficacy of subcutaneous AIT in horses with different allergic syndromes. Part II evaluates the occurrence and type of adverse events (AEs) associated with AIT in horses. This review was performed in accordance with the PRISMA (Preferred Reporting Items for Systematic Reviews and Meta-Analyses) guidelines [8].

### 2.1. Eligibility Criteria

#### 2.1.1. Part I—Efficacy of Subcutaneous Allergen-Specific Immunotherapy in Horses

We included articles that utilized allergen-specific immunotherapy formulated based on intradermal tests (IDT) or serology in naturally affected horses with respiratory or cutaneous allergic diseases and reported clinical outcomes. Clinical signs could manifest as allergic asthma (chronic cough, previously known as recurrent airway obstruction or chronic obstructive pulmonary disease), urticaria (pruritic or non-pruritic), or pruritus (with or without dermatitis, and with or without urticaria).

#### 2.1.2. Part II—Allergen-Specific Immunotherapy-Induced Adverse Events

To report the adverse events associated with AIT in horses, we included articles that specifically reported adverse events or the absence thereof. To provide data relevant to clinical practice, we excluded articles involving the use of recombinant allergens, prophylactic AIT, or inhalant AIT.

### 2.2. Search Strategy

Both authors searched three databases on September 18th, 2023, excluding review articles without any time restrictions and with the following strings:

1. PubMed: (horse* (tiab) or equine* (tiab) or “horses” (mesh)) AND (atop* (tiab) or allerg* (tiab) or hypersensitive* (tiab) or RAO(tiab) or COPD (tiab) or respirator* (tiab) or urticaria* (tiab) or hive* (tiab) or wheeze*(tiab) or asthma* (tiab) or “insect bite hypersensiti*” (tiab) or culicoide* (tiab))) AND (immunotherapy* (tiab) or desensiti* (tiab) or hyposensiti* (tiab) or hyposensitis* (tiab) or desensitis* (tiab) or “Desensitization, Immunologic” (mesh)).
2. Web of Science Core Collection: (horse* or equine*) AND (atop* or allerg* or hypersensitiv* or RAO or COPD or respirator* or asthma* or urticaria* or hive* or wheeze* or insect bite hypersensiti* or culicoide*) AND (immunotherap* or desensiti* or hyposensiti* or hyposensitis* or desensitis*).
3. CAB Abstract: (horse* or equine*) AND (atop* or allerg* or hypersensitiv* or RAO or COPD or respirator* or asthma*or urticaria* or hive* or wheeze* or insect bite hypersensiti* or culicoide*) AND (immunotherap* or desensiti* or hyposensiti* or hyposensitis* or desensitis*).

To increase the availability of evidence, we included reports in conference proceedings whenever sufficient relevant details were provided.

The bibliography of each selected article was subsequently screened for additional relevant articles.

### 2.3. Outcome Data

#### 2.3.1. Part I—Efficacy of Subcutaneous Allergen-Specific Immunotherapy in Horses

Primary outcome data evaluated were AIT-induced clinical remission and beneficial effects (see below for definition). Whenever an active and placebo group was available, the exact Fisher’s test was used to calculate a significant difference between the outcome of the two groups (https://www.socscistatistics.com/tests/fisher/; significance level 0.05; accessed on 15 June 2023). Secondary data are biases potentially affecting the study outcome. Each identified article was assessed by one author for analytical biases and confounding factors. Common study biases in AIT research assessed were study duration, concurrent use of anti-allergic medication, potential seasonal change influencing the clinical signs, and changes in the environment of the horses. Further data extraction included study subject specifics (breed, age, sex) and AIT characteristics (type, allergens, concentration, adjuvants, duration).

#### 2.3.2. Part II—Allergen-Specific Immunotherapy-Induced Adverse Events

Information selected from the articles reporting adverse events in horses treated with AIT were type and time of AE in relation to AIT administration. We report the AEs based on the grading system shown in Table 1. We modified the grading system proposed by the World Allergy Organization to characterize AEs that occur during AIT (Table 1) [9,10,11].

**Definition:**

**Beneficial effect:** A beneficial effect was defined as a reduction in clinical signs by equal to or greater than 50%, comparing the pre- and post-AIT scores during the allergy season. This information was either explicitly reported by the study or calculated by the authors based on individual outcome data available in the article.

**Clinical remission:** Clinical remission was defined as the absence of allergic symptoms during the allergy season without the need for anti-allergic medications (except AIT). This information was either reported explicitly by the study or individual clinical scores showed a lack of clinical signs.

**Outcome definition:** A detailed description of the clinical score was provided in the study that would be sufficient to reproduce the clinical assessment.

**Appropriate study duration:** For seasonal disease, an appropriate study duration was defined as the AIT being administered until at least the following season. For year-round clinical symptoms, a minimum of 9 months was considered appropriate.

**Allergen avoidance:** Allergen avoidance strategies included environmental changes such as increased stable time, as well as the use of repelling products of chemical and physical nature to prevent insect bites or limit contact with airborne allergens.

**Anti-allergic medication:** Glucocorticoids and antihistamines, whether administered orally or topically, were considered effective anti-allergic medications for controlling or preventing clinical signs of allergic diseases in horses [5,12]. Furthermore, oral fatty acids or topicals containing fatty acids were also considered as treatments, as studies have demonstrated their clinical efficacy or a positive change in the owner’s assessment of their horse’s clinical signs [13,14].

## 3. Results

### 3.1. Identified Evidence

**Figure 1:**
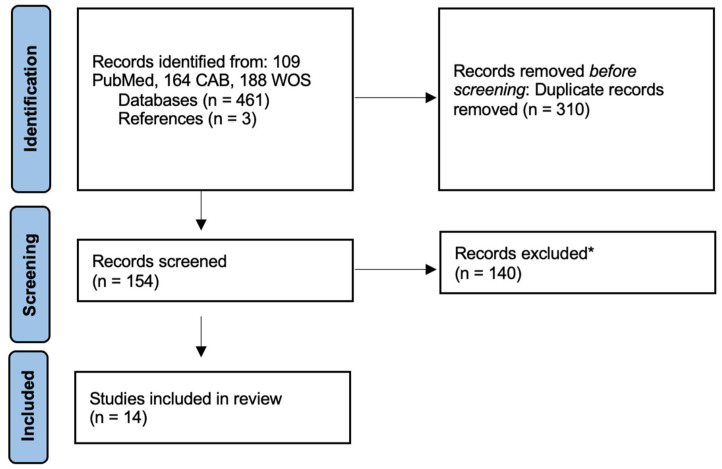
Flow diagram of literature search. * Records did not fulfill inclusion criteria.

Our search identified a total of 460 records across three databases. After screening, 14 articles were included in this review (Figure 1) [15,16,17,18,19,20,21,22,23,24,25,26,27,28].

Part I of the review focuses on the efficacy of AIT in horses with different allergic syndromes. The results are further grouped into AIT efficacy in respiratory diseases and cutaneous diseases. Cutaneous diseases encompass urticaria and pruritic dermatitis. The reviewed literature did not provide a standardized strict elimination criterion for insect bite hypersensitivity (IBH) prior to diagnosing seasonal atopic disease. Both diseases can present with overlapping clinical signs and co-sensitization is common [1,23]. Thus, we grouped horses with pruritic dermatitis together and focused the review on the allergen content in AIT (insect monotherapy and multi-allergen AIT containing allergens of multiple origins).

It is important to note that some articles included populations of horses with different clinical syndromes or subgroups treated with different allergen mixtures. As a result, the data from these articles were evaluated and presented separately, matching the respective categories [17,22,23,26].

Part II of the review addresses adverse events related to the administration of AIT and includes nine studies [15,18,19,22,23,24,25,26,27].

### 3.2. Part I Efficacy of Subcutaneous Allergen-Specific Immunotherapy

**A.**  **Efficacy of subcutaneous allergen-specific immunotherapy in respiratory diseases (allergic asthma)**

Five publications including 153 horses, treated with AIT for respiratory signs consistent with equine asthma were evaluated (Table 2) [15,16,17,21,22]. Detailed information pertaining to the studies can be accessed in Supplementary Table S1. One case series reported clinical remission in two horses while treated with AIT [21]. Among the 151 horses analyzed (excluding the 2 horses from the case series), a beneficial effect of AIT was reported in 114 horses (75%) presenting with respiratory signs (Table 2). A total of 6 of the 151 horses were observed to be free of clinical signs at the end of the study. In all five reports, AIT was formulated based on IDT results and contained various allergens including molds, insects, and environmental allergens with the exception of one horse. One study reported a 100% response in clinical signs to treatment with an only insect-containing AIT whereas the other two horses treated with a multi-allergen AIT showed only a 0% and 25% clinical response [17].

Several major limitations are associated with the findings of these studies. Firstly, the uncontrolled study designs and the presence of analytical and confounding biases could significantly impact the results. It is worth noting that two studies implemented environmental changes and allergen avoidance strategies concurrently with the AIT, which were not reported in the remaining three studies. Moreover, the short duration of the studies and the lack of information regarding the use of antiallergic medications (as shown in Table 2) pose further limitations.

Based on the published uncontrolled studies, AIT appears to provide beneficial effects for horses with respiratory diseases. However, biases leading to favorable results cannot be excluded, and it is crucial to conduct controlled studies in the future to accurately assess the true benefits of AIT while considering confounding factors.

**B.**  **Efficacy in immunological urticaria**

Three reports were included in the assessment, involving a total of 47 horses that received AIT for recurrent urticaria with or without pruritus and minor dermatitis (Table 3) [20,22,23]. Detailed information pertaining to the studies can be accessed in Supplementary Table S2. Across all reports, AIT contained various allergens including insect and environmental allergens. Overall, 88% of horses were reported to show a beneficial outcome while treated with AIT; excluding the case series, where a beneficial outcome of 100% is expected.

Within the case series, three horses demonstrated an excellent response, showing no recurrence of urticaria and requiring no additional medications [20]. The exact duration of treatment was not explicitly stated in two studies; however, it was noted that the average duration of treatment was equal to or greater than two years in the case series [20]. Consequently, the potential influence of seasonality on beneficial outcomes is limited in this study, but questionable in the remaining two studies. In addition, at least one of the studies initiated environmental changes at the beginning of the study. Overall, further limitations are the absence of a placebo group to show the true benefit of AIT in urticarial horses and the lack of reported medication scores in two studies.

In conclusion, although the number of included horses in the studies is small and confounding factors like allergen avoidance cannot be excluded, the published evidence suggests a beneficial effect of subcutaneous immunotherapy for horses suffering from urticaria.

**C.**  **Pruritus and dermatitis**

**CI. Pruritus and dermatitis treated with insect monotherapy**

Data from four studies were analyzed [17,24,25,26], focusing on the treatment of horses with pruritus and dermatitis using insect allergens exclusively, including two randomized controlled trials (RCTs) (Table 4) [24,26]. Detailed information pertaining to the studies can be accessed in Supplementary Table S3. The beneficial effects reported in the active treatment groups were found to be comparable to those in the placebo groups (Fisher’s exact test *p* = 0.5); and across all studies, less than half of the treated horses showed a beneficial response. Normal clinical scores were achieved in 6 of 28 horses (21%).

The studies reporting the efficacy of insect monotherapy AIT in horses display several major limitations and confounding factors that should be considered. Firstly, the low number of horses included in these studies. Secondarily, the potential influence of seasonal changes on the outcomes and the short duration of the two studies should be noted, as the follow-up period was only three or six months [24,26]. One study followed the horses over a single summer season [17], while another study [25] spanned two seasons. In the uncontrolled prospective study conducted by Anderson et al., a beneficial effect was reported in 5 out of 10 horses (50%), with a normal clinical score observed in 4 out of 10 horses (40%) at the end of the second season. However, this study and two additional ones do not report whether concurrent medications were permitted.

In conclusion, the evidence for the effectiveness of insect monotherapy is not supported by the evaluated studies. No significant difference was observed in the beneficial outcomes between the active and placebo groups and changes in the environment leading to improved scores in some horses cannot be excluded.

**CII. Pruritus and dermatitis treated with multi-allergen immunotherapy**

Seven studies were included in the evaluation, reporting the outcomes of horses treated with multi-allergen immunotherapy (AIT) for pruritic skin lesions (Table 5) [17,18,19,23,26,27,28]. Detailed information pertaining to the studies can be accessed in Supplementary Table S4. These studies encompassed a combination of pruritic dermatitis with our without urticaria and included two randomized controlled trials (RCTs) [26,27]. All studies used a mixture of environmental allergens (pollens, molds, and mites), and four studies also included insect allergens when reactions were positive on the IDT or serology [17,19,26,27].

A beneficial response was reported in 54 out of 91 horses (59%) receiving active AIT, compared to 1 out of 7 horses (14%) in the placebo group (Fisher’s exact test *p* = 0.04). However, it should be noted that the placebo group was small, and caution should be exercised in overinterpreting the results.

Notably, in one study [26], horses initially assigned to the placebo group were later switched to active treatment, thereby also being included in the active AIT group. Additionally, this study had a non-blinded part where horses previously treated with insect monotherapy were switched to receive 3 months of AIT containing multiple allergens based on the results of intradermal testing (IDT). Interestingly, the beneficial response was higher when horses initially treated with insect AIT for 3 months were crossed over into the multi-allergen AIT group. Out of the five horses that did not exhibit a beneficial response to insect monotherapy after 3 months, three horses achieved a beneficial response after 3 months of multi-allergen AIT. It is important to consider that the favorable response might have been influenced by the extended duration of treatment rather than the allergen content of AIT.

The second RCT conducted by Ginel et al. suggests a lack of effectiveness of insect-predominant, multi-allergen AIT. In this study, 10 horses with signs consistent with insect bite hypersensitivity were treated with AIT, predominantly targeting Culicoides allergens, including additional allergens such as arthropods, weed pollen, and mites in 7 out of 10 horses; therefore, the study was included in the multi-allergen analysis. No difference in response to AIT between the active and placebo groups was reported and none of the horses reached clinical remission. The study does not report individual outcomes and therefore, the results could not have been included in the analysis. It also remains unclear if these horses strictly suffered from insect bite hypersensitivity, as clinical lesions and pruritus seemed year-round with seasonal exacerbations based on the clinical scores provided.

Indeed, there are several limitations to consider when interpreting the results of the reviewed studies on multi-allergen AIT in horses. One major limitation is the variability in study designs, which can introduce bias and affect the robustness of the findings. Additionally, the small number of horses, particularly in the placebo group, reduces the statistical power and may limit the generalizability of the results.

Another limitation is the presence of confounding factors that can influence the treatment outcomes. Factors such as allergen avoidance and concomitant medication use during AIT were often not adequately reported in the studies, making it difficult to assess their impact on the treatment response.

The reported beneficial response to multi-allergen AIT ranging from 29% to 100% highlights the variability in treatment outcomes observed across the studies. It is important to note that the study reporting the lower response rate of 29% was conducted over a relatively short duration of 3 months, and longer treatment durations may yield higher response rates [26]. However, this emphasizes the need for prospective long-term studies to accurately evaluate the efficacy of AIT in horses.

Considering the limitations mentioned, it is crucial to interpret the results with caution and recognize the need for further research to address these limitations and provide more robust evidence on the efficacy and optimal use of multi-allergen AIT in horses.

### 3.3. Part II Subcutaneous Allergen-Specific Immunotherapy-Induced Adverse Events in Equines

The review of nine studies identified 35 reported adverse events (AEs) in horses receiving AIT (Table 6). Detailed information pertaining to the studies can be accessed in Supplementary Table S5. Local reactions, characterized by wheals, swelling, and pruritus at the injection site, were the most common AE (31/35) and affected 16–100% of horses across five studies. It is worth noting that these local reactions were described as self-limiting in four studies.

Two studies reported three horses experiencing systemic signs consistent with IgE-mediated reactions. One horse treated for pruritic dermatitis exhibited nervousness, sweating, and an urticarial reaction 15 min after the AIT injection. Another horse treated for urticaria experienced worsening of the urticaria 4 h after the first AIT injection. The third horse, suffering from severe urticaria and early asthma, developed angioedema of the hind limbs and sheath, which was noticed 24 h after the 14th injection. One study reported an increase in tube clotting time that was considered unlikely to be associated with AIT.

It is important to acknowledge the limitations of the available literature regarding AEs in AIT-treated horses. Many studies either did not mention AEs or did not provide detailed information on the likelihood of attributing the reactions to AIT. Some reports described AEs without specifying the number of affected horses, making it impossible to calculate the overall percentage of affected horses. In addition, administration errors like the unintentional intravenous injection of AIT by the owners/caregivers cannot be ruled out.

In conclusion, localized reactions at the injection site seem common in horses receiving AIT, but they are typically mild and self-limiting. However, serious and severe systemic reactions have been reported, and it is essential to discuss these potential risks with horse owners before initiating AIT. Based on the reviewed literature and the low number of horses reported having systemic reactions, it remains unclear if the risk of systemic reactions is associated with the specific disease that is treated with AIT, the allergy season, or the severity of clinical signs during treatment.

## 4. Discussion

This review evaluated the efficacy and safety of subcutaneous allergen-specific immunotherapy (AIT) in horses with various allergic syndromes in a systematic manner; concluding the efficacy of AIT depending on the specific disease that is treated and/or the allergen content in the AIT. Based on the available literature, horses with allergic asthma and urticaria seem to respond favorably to subcutaneous AIT, similar to horses diagnosed with pruritic dermatitis and treated with multi-allergen AIT.

Allergen-specific immunotherapy has been used for over a century to induce tolerance against allergens and to alleviate clinical signs [29]. AIT promotes a shift in the immune response from a T helper cell type 2 (Th2) phenotype, typically observed in allergic diseases, towards a Th1 and T regulatory immune response [30]. This immune modulation is achieved through various mechanisms, including the generation of blocking allergen-specific antibodies and the establishment of an anti-inflammatory cytokine milieu [30].

### 4.1. Part I—Efficacy of AIT

In this review, the outcome of horses with respiratory signs such as chronic cough, dyspnea, and asthmatic crisis, consistent with equine asthma (EA) treated with AIT was assessed. The severity of clinical signs and the content of the AIT varied among horses in the respiratory group, but overall, a beneficial effect was reported in the reviewed literature, ranging from 33% to 97% of treated horses. Interestingly, the exact role of allergic sensitization in EA remains unclear [2]. While an association between an increase in grass pollen and asthma exacerbations has been observed [31], EA is considered a multifactorial disease influenced by the individual genetic background, exposure to the environment, and potentially exacerbating infectious causes [2]. The extent to which the treatment effect of AIT in EA is due to true allergen desensitization or environmental changes is still uncertain, as specific information about the environmental modifications implemented in the studies reviewed was lacking. However, comparative analysis of AIT in respiratory diseases, including studies in human and feline allergology, suggests a specific effect of AIT in controlling clinical symptoms [32,33,34]. For example, allergen-specific immunotherapy is effective in managing symptoms of human asthma driven by house dust mites or pollen sensitization [32] and in cats with experimental asthma [33,34].

Future perspectives in the field of respiratory diseases include exploring local delivery of AIT through inhalation and combining it with cytosine–phosphate–guanine oligodeoxynucleotides (CpG-ODN) [35]. CpG-ODN alone is also being investigated as a potential allergen-independent anti-allergic immunomodulator [36].

Similar to equine asthma, AIT has shown clinical benefit in the treatment of immunologically mediated urticaria in horses. The formation of wheals and angioedema is considered a syndrome and can have multiple etiologies, some immune-mediated and others non-immunologically (e.g., heat, exercise, etc.) [37,38]. Allergens reported to trigger chronic urticaria (CU) in horses are of food, insect, or environmental origin [1]. Noteworthy is that CU in people is often caused by autoimmunity towards the IgE and/or IgE receptors where allergen-specific desensitization is not recommended [39]; however, studies have shown a beneficial effect in a small number of people with immunological urticaria sensitized to allergens [40].

Interestingly, the efficacy of multi-allergen AIT in horses with pruritic dermatitis overall is 59% (range 29–100%), comparable to the results of dogs with atopic dermatitis (AD) and cats with allergic skin syndrome treated with AIT [41,42]. However, the adequate diagnosis of horses with atopic dermatitis, especially in areas where the season of biting insects overlaps with the grass and weed pollen season, is still unclear. In addition, food allergies can be difficult to diagnose and manage in horses. The diagnoses of an environmental allergy would rely on strict insect prevention and the exclusion of food. In addition, many horses show polysensitization to various insect and environmental allergens in allergy tests [27,43]. It is unclear if this is only sensitization due to constant exposure or sensitization causing a mixture of clinical signs.

Within the reviewed studies, the inclusion criteria for horses with insect bite hypersensitivity and atopic dermatitis are heterogeneous and not standardized. The authors used a combination of clinical signs, including seasonality, lesion type and location, exclusion of other differentials with various means, and sometimes even strict sensitization patterns to differentiate IBH from seasonal AD. The overlap of seasonality in horses with pollen-sensitized atopic dermatitis and IBH, and the co-sensitization of horses to both insect and environmental allergens seem to make the strict diagnoses of atopic dermatitis or Culicoides hypersensitivity somehow artificial. Thus, we grouped horses with pruritic dermatitis together and focused the review on the allergen content in AIT to draw conclusions.

Of note is the lack of effect of insect monotherapy in pruritic horses. The immune response in IBH is of Th2 type and in theory, the mechanism of AIT would be effective. The lack of effect could be the allergen itself, not being able to induce a strong enough immune response. One of the reviewed studies combined the Culicoides allergens with strong adjuvants and Th1 inducer mycobacterial cell wall. Interestingly, this study reported the highest number of horses (50%) having a beneficial response. Low cross-reactivity between insect allergens and unrelated allergen groups such as mites and pollen was shown in horses [44]. This suggests that the possibility of true polysensitization in horses may require the inclusion of non-insect allergens in the allergen-specific immunotherapy (AIT) regimen to induce tolerance.

In one of the reviewed studies [26], five horses showing positive skin reactions to both insect and pollen allergens were initially treated with insect monotherapy for a duration of three months. Subsequently, these horses were switched to receive a multi-allergen AIT regimen for three months based on the results of intradermal testing (IDT) and showing a better response. These findings suggest that including multiple allergens in the AIT regimen may lead to improved clinical outcomes in horses with positive skin reactions to both insect and pollen allergens. It is important to note that this study involved a small population of horses and had a relatively short treatment duration of three months for each AIT regime. Furthermore, the specific season during which the clinical improvement was assessed was not reported, which could have influenced the results. Further research with larger populations and longer treatment durations, while taking into account the seasonal variations, would be valuable to validate these findings and provide more conclusive evidence. New therapies like allergen vaccines, new adjuvants, recombinant allergens, and targeting of the involved cytokines are currently explored in IBH [45,46].

### 4.2. Part II—Safety of AIT

Overall, allergen-specific immunotherapy (AIT) is generally considered safe for use in horses, but it is important to note that severe systemic reactions or anaphylaxis have been reported. In our analysis, we found that self-limiting local reactions were common in horses and affected a varying percentage of treated individuals, ranging from 16% to 100%. Similarly, local reactions are commonly observed in humans undergoing subcutaneous AIT, but they are rarely reported in dogs and cats [6,11,47]. One possible explanation for this difference in reporting between species could be the reduced attention given by owners to detect and report local reactions in animals with longer coats, such as dogs and cats. In our review, three horses were reported with systemic signs consistent with a grade II reaction. In human allergology, the presence of severe and uncontrolled allergic asthma as well as seasonal exacerbations are known to be major risk factors for severe reactions to AIT [48]. It is important to note that in the case of horses, one of the reported cases of systemic reaction to AIT involved a horse being treated for pruritic dermatitis, while the other two horses had urticaria, with one of them also presenting early asthma symptoms [22,24]. Unfortunately, detailed information regarding the timing of these reactions in relation to the treatment period and the overall control of the horses’ underlying diseases was not available. Therefore, the presence or absence of these risk factors in the reported cases cannot be definitively determined. In addition, administration errors like the unintentional intravenous injection of AIT by the owners/caregivers cannot be ruled out. In the field of human and canine allergology, sublingual immunotherapy (SLIT) is generally considered to have a safer profile compared to subcutaneous injections [49,50]. Interestingly, there was a case of a horse treated with SLIT for seasonal pruritus and urticaria that experienced a grade III systemic reaction characterized by scleral edema, moderate dyspnea, and edema of the mammary gland [51]. This highlights the importance of owner education and close monitoring for all forms of AIT applications in horses to ensure early detection and appropriate management of potential adverse reactions.

### 4.3. Limitations of the Reviewed Literature

There are several limitations that should be considered when evaluating the results of this review. Firstly, among all the studies that assessed the efficacy of AIT, only three out of thirteen were randomized controlled trials (RCTs), while four were prospective studies, three were retrospective studies, and two were case series. It is important to note that studies providing stronger evidence, such as RCTs or prospective studies, were distributed across all the evaluated subgroups in a balanced manner. Analytical biases in this analysis arise from the heterogeneity of study designs, inclusion criteria, and the use of non-standardized outcome scores. The variability in these factors can introduce potential sources of bias and limit the comparability of the results across studies. Furthermore, it is crucial to acknowledge that the publications included in this review span a period of four decades. During this time, there have been significant advancements in the understanding of allergic diseases and the quality of allergen extracts used for AIT. Therefore, the older studies may not reflect the current standards in the field.

In this review, we employed a criterion of ≥ 50% reduction in clinical signs to define a beneficial effect, as this was the most utilized outcome measurement in the literature. However, the use of this measurement as a meaningful clinical parameter for disease control and patient and owner satisfaction remains debatable. It is important to consider that even with a beneficial reduction in clinical signs, a high level of pruritus may persist, necessitating the continued use of anti-allergic medications to manage symptoms. Proposed outcomes for therapeutic trials in allergic diseases suggest assessing the reduction of investigator- and owner-assessed clinical parameters, such as skin lesions and pruritus scores, to a level of normal to mild severity [52]. Therefore, in our analysis, we also considered clinical remission as a meaningful clinical outcome. Unfortunately, the majority of studies did not report this information or provide individualized outcome data for analysis. Another significant aspect to note is that the studies included in this review often utilized whole extracts of allergens, and in some cases, these extracts were self-prepared due to the lack of commercially available allergens at the time of the studies. The lack of standardization in the allergen content of commercially available solutions is a notable limitation within the field.

In addition to the identified analytical biases, the potential influence of confounding factors on the outcomes of the reviewed studies was evaluated. One notable finding was the lack of reporting on concurrent medication use. Only one out of seven studies that stated medication use was allowed provided information on individual medication scores [20]. It is important to consider that the use of anti-allergic medication, including topical products such as shampoos, can alleviate clinical signs and potentially impact owner-assessed outcome scores [13]. In the equine field, the widespread use of over-the-counter nutritional supplements and various topical treatments further complicates the control of these interventions, which may have influenced the clinical outcomes.

Furthermore, our objective was to elucidate outcome biases by examining allergen-avoidance strategies implemented during the study periods in the reviewed literature. We regarded an increase in stable time as a pivotal factor for preventing insect bites, which subsequently contributed to an amelioration of clinical signs in horses sensitized to these allergens. Nevertheless, it is noteworthy that indoor housing may inadvertently elevate allergen exposure for horses sensitized to house dust mites or molds.

This systematic review aims to provide an overview of the existing information regarding subcutaneous allergen-specific immunotherapy (AIT) in horses with allergic diseases. The included studies in this review display heterogeneity in terms of study design, analytical methods, and the presence of confounding factors. While there are uncertainties in the available evidence, AIT is frequently utilized in horses with allergic conditions when other approaches, such as insect avoidance, fail to yield substantial clinical improvement, and it is believed to be effective. Larger controlled studies are needed to establish the true effect of AIT while controlling for confounding factors. The challenges associated with studying AIT in horses include the lack of standardized diagnostic approaches and clinical scoring systems, as well as the extended study duration required. Furthermore, advances in allergology, such as the use of allergoids, recombinant allergens, plasmid vaccines, and specific adjuvants, are shaping the field and may lead to more targeted immune responses [29,35,53]. Until these advancements become commercially available, practitioners will continue to rely on conventional allergen-specific immunotherapy, and the findings of this review can aid in client education regarding efficacy and safety.

## 5. Conclusions

The findings of this systematic review underscore the current limitations in the available literature, highlighting the paucity of robust evidence and the presence of various analytical and confounding biases. Consequently, the interpretation of the results from these studies presents a notable challenge. Summarizing the evidence, multi-allergen AIT appears to yield positive outcomes in horses afflicted with respiratory diseases, urticaria, and pruritic dermatitis, whereas the efficacy of insect AIT for pruritic horses does not appear to be favorable. Self-limiting local reactions were the most common adverse events associated with AIY in horses, often manifesting as injection site reactions. To advance our understanding of the efficacy and safety of AIT in equine patients suffering from allergic diseases, further research is imperative. Such investigations should aim to limit biases and enhance the quality of evidence available in this field.

## Figures and Tables

**Table 1:** Grading system for allergen immunotherapy (AIT)-induced local and systemic adverse events (AEs) in animals, modified from [9,10].

Local Adverse Event (LR)	Systemic Adverse Event (SR)		
	Grade I	Grade II	Grade III
Localized cutaneous reaction	One organ system affected	More than 1 organ system affected; OR	Severe anaphylaxis
	**Cutaneous**	**Cutaneous**	**Upper respiratory**
Localized pruritus at injection site	Generalized pruritus	Urticaria, flushing, angioedema (not oral)	Laryngeal, tongue edema
or	or	or	or
Swelling of injection side	**Upper respiratory**	**Lower respiratory**	**Lower or Upper respiratory**
	Rhinitis	Cough, wheezing	Respiratory failure
or	or	or	or
Infection/abscess at injection side	Cough		
	or		
	**Conjunctival**	**Gastrointestinal**	**Cardiovascular**
	Erythema, pruritus, tearing	Vomiting, diarrhea	Hypotension, hypertension
	or		
	**Other**		
	Lethargy, anorexia, nausea		

**Table 2:** Summary of studies using subcutaneous AIT in horses with respiratory signs.

						Analysis Biases		Confounding Biases	
Author [Reference]	Year	Study Design	Number of Horses	Beneficial Outcome	Clinical Remission	Outcome Definition Reported	Appropriate Observation Period	Allergen Avoidance Implemented	Medications Allowed/ Reported
Carr [21] ^1^	1978	Case series	2	2 (100%)	2 (100%)	No	Yes	NR	NR
Beech [15]	1986	Retrospective	99	66 (67%)	NR	Yes	NR	Yes	Yes/no
Francqueville [16]	1989	Prospective uncontrolled	13	12 (92%)	5 (38%)	Yes	NR	NR	NR
Fadok [17]	1996	Prospective uncontrolled	3	1 (33%)	1 (33%)	Yes	Yes	NR	NR
Tallarico [22]	1998	Prospective uncontrolled	36	35 (97%)	NR	Yes	No	Yes	Yes/no
**Beneficial outcome**				**114/151 (75%) ^1^**					

^1^ Data of case series not included in analysis; NR: not reported.

**Table 3:** Summary of studies using subcutaneous AIT in horses with urticaria.

						Analysis Biases		Confounding Biases	
Author [Reference]	Year	Design	Number of Horses	Beneficial Outcome	Clinical Remission	Outcome Definition Reported	Appropriate Observation Period	Allergen Avoidance Implemented	Medications Allowed/ Reported
Tallarico [22]	1998	Prospective uncontrolled	14	14 (100%)	NR	Yes	NR	Yes	Yes/no
Rees [20] ^1^	2001	Case series	5	5 (100%)	3 (60%)	No	Yes	NR	Yes/yes
Stepnik [23]	2011	Retrospective	28	23 (82%)	NR	No	NR	NR	Yes/no
**Beneficial outcome**				**37/42 (88%) ^1^**					

^1^ Data of case series not included in analysis; NR: not reported.

**Table 4:** Summary of studies using exclusively insect allergens in subcutaneous AIT in horses with pruritic dermatitis and pruritus.

						Analysis Biases		Confounding Biases	
Author [Reference]	Year	Design	Number of Horses	Beneficial Outcome	Clinical Remission	Outcome Definition Reported	Appropriate Observation Period	Allergen Avoidance Implemented	Medications Allowed/Reported
Barbet [24] ^1^	1990	RCT: active arm	6	1 (17%)	0	Yes	No	Yes	Yes/no
		Placebo arm	7	1 (14%)	0				
Anderson [25]	1996	Prospective uncontrolled	10	5 (50%)	4 (40%)	Yes	Yes	No	NR
Fadok [17]	1996	Prospective uncontrolled	5	1 (20%)	1 (20%)	Yes	Yes	NR	NR
Rosenkrantz [26] ^2^	1996	RCT: active arm	7	3 (43%)	1 (14%)	Yes	No	NR	NR
		Placebo arm	6	2 (33%)	0				
**Beneficial outcome AIT**				**10/28 (36%)**					
**Beneficial outcome placebo**				**3/13 (23%)**	Fisher’s exact test *p* = 0.5				

^1^ Outcome was assessed for analysis by comparing pre-trial June clinical scores with September clinical scores (peak of season), ^1^ horse 4 in placebo group was excluded as no clinical signs were present at the beginning of the study, ^2^ one horse in placebo group was excluded due to only pretest clinical score was available; NR: not reported.

**Table 5:** Summary of studies using subcutaneous multi-allergen AIT in horses with pruritic dermatitis and pruritus.

						Analysis Biases		Confounding Biases	
Author [Reference]	Year	Design	Number of Horses	Beneficial Outcome	Clinical Remission	Outcome Definition Reported	Appropriate Observation Period	Allergen Avoidance Implemented	Medications Allowed/ Reported
Fadok [17]	1996	Prospective uncontrolled	17	12 (71%)	4 (24%)	Yes	Yes	NR	NR
Rosenkrantz [26] ^1^	1996	RCT: active	7	2 (29%)	0	Yes	No	NR	NR
		RCT: placebo	7	1 (14%)	0				
		Non-blinded	7 ^1^	3 (43%)	1 (14%)				
		Cross-over, non-blinded	5	4 (80%)	0				
Stepnik [23]	2011	Retrospective	4	4 (100%)	NR	No	NR	NR	Yes/no
Ginel [27]	2014	RCT: active	5	NR	0	Yes	Yes	Yes	No
		RCT: placebo	5	NR	0				
Loeffler [18]	2018	Retrospective	14	9 (64%)	NR	No	NR	Yes	Yes/no
Martels [28]	2019	Prospective uncontrolled	27	15 (56%)	NR	Yes	Yes	Yes	No
Radwanski [19] ^2^	2019	Prospective uncontrolled	17	8 (47%)	3 (18%)	Yes	Yes	Yes	Yes/no
**Beneficial outcome of AIT**				**54/91 (59%)**					
**Beneficial outcome placebo**				**1/7 (14%)**	Fisher’s exact test *p* = 0.04				

^1^ Same seven horses reported in the active study arm were included in non-blinded evaluation after 6 months of AIT, the non-blinded data were excluded from analysis; ^2^ two horses were excluded due to missing outcome information. NR: not reported.

**Table 6:** Summary of AIT-induced adverse events (AEs) reported in the reviewed literature. NR: not reported; SR: systemic reaction.

Author [Reference]	Year	Number of Horses	Clinical Presentation	Total AEs	Local Reaction	Systemic Reaction	SR Grade I	SR Grade II	Reaction
Beech [15]	1986	99	Asthma	NR	NR	0	0	0	Few subcutaneous firm swellings at injected site
Barbet [24]	1990	21	Pruritic dermatitis	NR	NR	NR	NR	1 (5%)	Nervous sweating, urticaria, and rapid respirations
Anderson [25]	1996	10	Pruritic dermatitis	10 (100%)	10 (100%)	0	0	0	Local wheal and local pruritus
Rosenkrantz [26]	1996	27	Pruritic dermatitis	6 (22%)	6 (22%)	0	0	0	Swelling at injection site reaction ranging from few to several centimeters, self-limiting in 24–72 h
Tallarico [22]	1998	50	Asthma or urticaria	NR	NR	NR	NR	2 (4%)	Swelling at the injection site for 24–36 h, increase in urticaria, angioedema of hind limbs
Stepnik [23]	2011	32	Pruritic dermatitis	5 (16%)	5 (16%)	0	0	0	Swelling at injection site, regression within 24–48 h without medications
Ginel [27]	2014	20	Pruritic dermatitis	0	0	0	0	0	Na
Loeffler [18]	2018	14	Pruritic dermatitis	6 (43%)	6 (43%)	0	0	0	Self-resolving swelling at the injection site
Radwanski [19]	2019	19	Pruritic dermatitis +/- urticaria	5 (26%)	4 (21%)	0	0	0	Local reaction site reaction responding to dose adjustments, one horse reported prolonged tube clotting times during blood sampling
**Total AIT-induced AEs**				**32/122 (26%)**					

## Data Availability

Newly generated data (reanalyzed from original work) are contained within the article or supplementary material. The data presented in this study are available in (insert Supplementary Materials S1–S5).

## Supplementary Materials

The following supporting information can be downloaded at: https://www.mdpi.com/article/10.3390/vetsci10100613/s1, Table S1: Detailed information of studies reporting horses treated with subcutaneous allergen-specific immunotherapy for respiratory diseases; Table S2: Detailed information of studies reporting horses treated with subcutaneous allergen-specific immunotherapy for urticaria; Table S3: Detailed information of studies reporting horses treated with subcutaneous allergen-specific immunotherapy solely containing insect allergens for pruritic dermatitis; Table S4: Detailed information of studies reporting horses treated with subcutaneous multi-allergen immunotherapy solely containing insect allergens for pruritic dermatitis; Table S5: Detailed information of studies reporting AIT-induced adverse events in horses.
